# The Crosstalk between Nerves and Cancer—A Poorly Understood Phenomenon and New Possibilities

**DOI:** 10.3390/cancers16101875

**Published:** 2024-05-15

**Authors:** David Benzaquen, Yaacov R. Lawrence, Daniel Taussky, Daniel Zwahlen, Christoph Oehler, Ambroise Champion

**Affiliations:** 1 Radiation Oncology, Hôpital de La Tour, 1217 Meyrin, Switzerland; david.benzaquen@latour.ch (D.B.); ambroise.champion@latour.ch (A.C.); 2 Department of Radiation Oncology, Sheba Medical Center, Tel-Aviv 39040, Israel; yaacov.lawrence@sheba.health.gov.il; 3 Department of Radiation Oncology, Centre Hospitalier de l’Université de Montréal, Montréal, QC H2X 0C1, Canada; 4 Department of Radiation Oncology, Kantonsspital Winterthur, 8400 Winterthur, Switzerland; daniel.zwahlen@ksw.ch (D.Z.); christoph.oehler@ksw.ch (C.O.)

**Keywords:** cancer, nerves, tumor microenvironment, axonogenesis

## Abstract

**Simple Summary:**

There is an interaction between cancer and neural cells. This interaction was discovered as early as 1948. The first messenger of this interaction, called crosstalk, was discovered shortly after and was named the nerve growth factor (NGF). This factor influences many different organs, among them, the immune system and the nervous system. Its influence on cancer is little-known. We summarized the present knowledge on the interaction between cancer and nerves with a special focus on prostate and pancreas cancer, cancers that are richly innervated and therefore called neurotrophic. We found that this interaction plays a crucial role as a regulator of the cancer microenvironment. The surgical, chemical, and radiotherapeutical ablation of nerves has been shown to have a therapeutic effect on both cancers. There seems to be a potential relationship between cancer and psychosocial biology via neurotransmitters and neurotrophins such as the NGF. The effect of beta-blockers on cancer seems limited so far.

**Abstract:**

Introduction: Crosstalk occurs between nerve and cancer cells. These interactions are important for cancer homeostasis and metabolism. Nerve cells influence the tumor microenvironment (TME) and participate in metastasis through neurogenesis, neural extension, and axonogenesis. We summarized the past and current literature on the interaction between nerves and cancer, with a special focus on pancreatic ductal adenocarcinoma (PDAC), prostate cancer (PCa), and the role of the nerve growth factor (NGF) in cancer. Materials/Methods: We reviewed PubMed and Google Scholar for the relevant literature on the relationship between nerves, neurotrophins, and cancer in general and specifically for both PCa and PDAC. Results: The NGF helped sustain cancer cell proliferation and evade immune defense. It is a neuropeptide involved in neurogenic inflammation through the activation of several cells of the immune system by several proinflammatory cytokines. Both PCa and PDAC employ different strategies to evade immune defense. The prostate is richly innervated by both the sympathetic and parasympathetic nerves, which helps in both growth control and homeostasis. Newly formed autonomic nerve fibers grow into cancer cells and contribute to cancer initiation and progression through the activation of β-adrenergic and muscarinic cholinergic signaling. Surgical or chemical sympathectomy prevents the development of prostate cancer. Beta-blockers have a high therapeutic potential for cancer, although current clinical data have been contradictory. With a better understanding of the beta-receptors, one could identify specific receptors that could have an effect on prostate cancer development or act as therapeutic agents. Conclusion: The bidirectional crosstalk between the nervous system and cancer cells has emerged as a crucial regulator of cancer and its microenvironment. Denervation has been shown to be promising in vitro and in animal models. Additionally, there is a potential relationship between cancer and psychosocial biology through neurotransmitters and neurotrophins.

## 1. Introduction

There is a bidirectional interaction between nerve cells and cancer cells. This interaction is critical for homeostasis and metabolism [1]. Nerve fibers and neural cells also play an important role in the tumor microenvironment (TME) [2].

Neurogenesis, neural extension, and axonogenesis are routes for cancer cell dissemination [3]. Neurogenesis, angiogenesis, and immune reactions can be manipulated by cancer for growth and metastasis [4]. Axonogenesis is the enlargement of nerves or an increase in nerve density (axon extension and number). Neurogenesis refers to an increase in the number of neurons [5].

The TME contributes to cancer-associated nerve networks. These networks are important for regulating tumorigenesis and metastasis and can exhibit axonal outgrowth.

The mechanism by which this crosstalk occurs between the central nervous system and cancer is not yet fully understood. It is known that there is a migration of central neural progenitors that nurture primary cancer development and metastasis. This shows that communication between cancer cells and distant organs is required to recruit cells for their growth and dissemination [1].

Some of the transmitters involved in this crosstalk are known. Neurochemicals, such as glutamate, norepinephrine, and acetylcholine, are secreted factors such as the nerve growth factor (NGF) [6]. The NGF is a neurotrophin and a brain-derived neurotrophic factor (BDNF) [7].

In a “*Roadmap for the Emerging Field of Cancer Neuroscience*” [8], Monje et al. stated that the crosstalk between the nervous system and cancer, both locally and systemically, is emerging as a crucial regulator of cancer initiation and progression. Neurotransmitters derived from peripheral nerves and growth factor signaling can regulate the progression of several cancers, including pancreatic ductal adenocarcinoma (PDAC) and prostate cancer (PCa), which are considered “neurotropic” cancers. They have been shown to be infiltrated by autonomic and sensory nerve fibers that release neurotransmitters [3]. Both exhibit perineural invasion (PNI). PNI is defined as a neoplastic invasion of nerves and is associated with tumor recurrence and pain [9]. PNI is associated with worse outcomes [10]. Ayala et al. [11] argued that the PNI is a reaction to the recruitment of neurites by cancer.

In the present study, we summarized the past and current literature on the history of the interaction between nerves and cancer, with a special focus on pancreatic ductal adenocarcinoma (PDAC), prostate cancer (PCa), and the role of the NGF in cancer.

## 2. Materials/Methods

We reviewed PubMed and Google Scholar for the relevant literature on the relationship between nerves, neurotrophins, and cancer in general and specifically for both PCa and PDAC. 

## 3. Results

### 3.1. A Short Historical View of the Interaction between the NGF and Cancer

The importance of the interaction between the nervous system and cancer was discovered in 1948 by Bueker [12]. They reported that peripheral nerves innervated a transplant of a mouse sarcoma into a chick embryo and that the nearby dorsal root ganglia started to become enlarged. Subsequently, in 1952, Levi-Montalcini proved that cancer released a humoral factor that gained access to the ganglia through the circulatory system. This factor was later identified and named as the nerve growth factor (NGF), for which she and S Cohen merited the Nobel Prize in 1986 [13,14,15]. More specifically, Levi-Montalcini discovered, in the laboratory of V. Hamburger in St Louis, Missouri, that limb bud extirpation in an embryo causes an increase in neuronal death. She realized that there must be a feedback mechanism between developing neurons and peripheral tissues. In 1950, she discovered the NGF as the first such factor [16]. Furthermore, since then, she observed that nerve fibers grow into cancer and that the size of the sensory ganglia that invade cancer increases compared to the contralateral non-invading ganglia. Therefore, in 1952, Levi-Montalcini proposed the hypothesis that cancer releases a humoral factor that gains access to the ganglia through the circulatory system [17,18]. Additionally, it was observed that nerve fibers branch into cancer without any direct contact with the cancer. All this led to an early interest in the interaction between cancer and nerves. It seems that Levi-Montalcini did not show much interest in the importance of the NGF in oncology. In her article titled “*The Nerve Growth Factor: Thirty-Five Years Later*” [13], she mainly talked about the potential of the NGF in neuroembryology, the general field of neuroscience, and its role in the immune system.

As this review illustrates, there is still little known about this interaction between the NGF and cancer. We are still trying to put the puzzle pieces together to obtain a broader picture of this crosstalk and how it can be used to explain cancer growth and use the knowledge to develop cancer therapies [16].

The NGF is a polypeptide. It can be extracted mainly from the mouse submandibular gland, and its concentration is much higher in male mice. This difference between male and female mice seems to be due to testosterone, which leads to an increase in the NGF [19]. It was later discovered that the NGF protein is part of a precursor and that the processor enzyme remains associated with the NGF to form a large, multimolecular complex defined as 7S NGF [20]. The NGF was the first identified factor to belong to the class of cell regulators. Because of its activity in both cancer and nerves, the NGF was first called nerve growth-promoting activity. The similarities of the NGF to insulin led to the classification of the NGF as an endocrine- or hormonal-like substance. Until then, endocrine substances were disseminated in the organism through the bloodstream, and the NGF levels in the blood were very small. Other arguments for the endocrinal nature of the NGF were the identification of NGF receptors mainly on neurons and that the NGF was synthesized like many other hormones as a precursor that is cleaved either intracellularly or extracellularly [18].

### 3.2. The NGF’s Receptors and Its Effect on Neurons

The NGF is the product of a single gene on chromosome 1. Tropomyosin-related kinase (Trk) receptors have a high affinity for the NGF [21]. Through the TrkA receptor, the NGF regulates neuronal cell survival and differentiation and axonal and dendritic growth. It also regulates synaptic formation and plasticity. The NGF acts as a protector of degenerating peripheral nerve cells and as a regulator of neurotransmitters and neuropeptides as well as on the synthesis of sympathetic and sensory nerve cells [22]. Peripheral nerve lesions cause NGF downregulation. The NGF was first thought to act only on neurons; later, it was discovered that it acts on different leukocytes and the endocrine system, and it is important for several homeostatic processes. Besides its effect on the nervous system, the NGF has an effect on epidermal wound healing and on the eye. This underlines the therapeutic potential of the NGF for several neurological diseases, such as Parkinson’s or Alzheimer’s, as well as ocular disorders, such as corneal ulcers and macular degeneration and wound healing in pressure ulcers [22]. The above-mentioned research has led to the hypothesis that because neurotrophins could stimulate cancer, the Trk receptor expression level could be a biomarker for different cancers [23,24]. TrkA is not only found in neuronal tissue but also in other cancers, such as breast cancer. Therefore, there is significant interest in the antibodies that target TrkA/NGF as a treatment for various cancers [25]. Neurotrophic tyrosine receptor kinase (NTRK) is a target for the treatment of many different cancers, mainly colon cancers but also other cancers, such as other gastrointestinal, gynecological, thyroid, lung, and pediatric malignancies [26]. Obviously, one could assume that the NGF might play a role in brain cancers. The NGF is expressed in glioblastoma multiforme (GBM), the most common primary malignant brain tumor in adults, and has been shown to stimulate GBM cells. Marsland et al. [21] found that the precursor for NGF, ProNGF, was overexpressed in GBM and that blocking it inhibited cell growth both in vitro and in vivo in humans. The value of ProNGF as a prognostic marker or therapeutic target is unknown.

### 3.3. The Crosstalk between Nerves and Cancer

This crosstalk between the nervous system and cancer occurs both through direct nerve–cancer interactions and via the nervous system regulation of other cell types within the tumor microenvironment, such as immune and endothelial cells. Table 1 summarizes the evidence of an interaction between cancer and nerves. Mauffrey et al. [1] showed that there are two neurogenic regions of the central nervous system that may be stimulated by cancer during its development to provide neural progenitors that initiate neurogenesis: the subventricular zone (SVZ) and the dentate gyrus in the hippocampus. These two centers may be stimulated by cancer during its development to provide neural progenitors that initiate neurogenesis.

**Figure 1:**
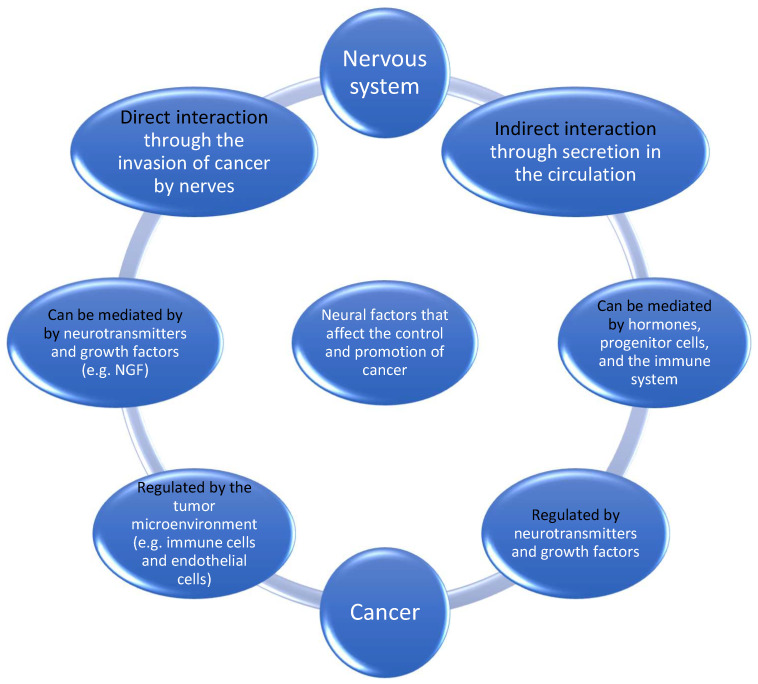
Neural factors that affect the control and promotion of cancer.

Hanahan and Monje [29], in their review on the intersection between cancer hallmarks and neuroscience, described that there is a participation of the nervous system in tumor-promoting inflammation. Hallmarks are defined as “a set of functional capabilities acquired by human cells as they make their way from normalcy to neoplastic growth states” [29]. Furthermore, neuronal signaling circuits in cancer cells are co-opted as regulatory mechanisms that modulate the development and malignant progression of at least some cancer subtypes. They believed that the nervous system has multiple connections with cancer. Figure 1 illustrates the interaction between nerve cells and cancer. One could argue that the crosstalk could be linked to the fact that some cancer types show a propensity for brain metastasis [30].

The depletion of neurogenic niches in the brain by attracting neural progenitors to support its own development might explain the cognitive impairment in cancer patients [1].

### 3.4. What We Know Now about the Interaction between the NGF and Cancer

The NGF can stimulate γ-aminobutyric acid (GABA) synaptogenesis [31]. GABA signaling can help sustain cancer cell proliferation and help in evading the immune defense. Moreover, it modulates tumor promotion, therefore balancing the defense against the immune response and T-cell attack [32].

There are different neutralizers or antibodies targeting the NGF pathway that have been tested in animals [33]. The surgical and pharmacologic tumor denervation of solid cancers resulted in the slowing or even stopping of cancer growth in mice [34].

### 3.5. A Common Feature in Both PCa and PDAC

Both are comparatively refractory to immunotherapy. It is known that PDAC employs different strategies to evade the immune defense [35]. Both are considered low immune-reactive cancers that represent either the limited infiltration of immune cells or the extensive infiltration of immunosuppressive T cells [36]. Pancreatic cancer has high cellular plasticity, meaning that the cancer cells can change and adapt to different microenvironments [37].

### 3.6. The NGF, the Immune System, and Inflammation

Early on, it was shown that the human innate immune response can be regulated by the NGF. The influence of the NGF on the immune system and its pathophysiology is multifaceted. In their editorial, Krita et al. [38] stated that the NGF is a neuropeptide involved in neurogenic inflammation through the activation of several cells of the immune system via several proinflammatory cytokines. The NGF is produced by the thymus and CD4+ T cells. The NGF induces T-cell maturation during infection. In B cells, the NGF stimulates B cell proliferation, antibody production, memory B cell survival, and CD40 expression [39]. During inflammation, mast cells release the NGF in high concentrations, leading to axonal outgrowth and causing elevated pain perception, while at the same time, the NGF activates pathways to attenuate the inflammatory response and reduce tissue damage. Genetic mutations affecting the production of the NGF or mutations in the tropomyosin receptor kinase (TrkA) receptors might be implicated in autoimmune diseases. Elevated NGF levels have been found in the blood and tissues of patients with several autoimmune diseases, including, among others, multiple sclerosis, systemic lupus erythematosus, autoimmune thyroiditis, and chronic arthritis [40]. This seems to make anti-NGF therapies very promising. However, disappointingly, the depletion of the NGF has led to neurodegenerative diseases and symptoms. The NGF is also involved in the production of immunoglobulins and can induce interleukin (IL)-2 receptors in human peripheral blood mononuclear cells and promote human hematopoietic cell growth and proliferation. The NGF is expressed in immune cells such as T and B lymphocytes, dendritic cells, and monocytes/macrophages [40]. The NGF is upregulated in inflamed tissues in many diseases and is associated with inflammatory pain. The interaction between the nervous and immune systems is not surprising, as both are implicated in homeostasis and adaptation to the environment [41].

This interaction between the NGF and the immune system has been illustrated in a recent study. In the tumor tissues of patients with hepatocellular carcinoma, it was found that T cells invading the microenvironment bound to the largest part of the NGF secreted by cancer cells. This caused a decrease in the communication between nerves and cancer [42].

### 3.7. The NGF and Pain

Inflammatory mediators, such as the nerve growth factor (NGF), can lower the activation thresholds of sensory neurons [43]. The NGF is considered a key contributor to pain [44]. The importance of the NGF in pain is well-recognized. Interestingly, TrkA plays an important role in nociception, nervous system plasticity, and pain [45]. The NGF is upregulated in patients with chronic pain syndromes. Patients with inflammatory and neuropathic pain have been shown to have increased NGF levels [46]. There are several clinical trials on humanized anti-NGF monoclonal antibodies (mAbs) as potential analgesic agents. The US Food and Drug Administration (FDA) stopped all clinical trials with anti-NGF monoclonal antibodies (mAb) in late 2010 because of serious joint-related adverse events and safety issues for the sympathetic nervous system. So far, the best results were found in patients with osteoarthritis of the knee and/or hip [47].

### 3.8. Can Nerve–Cancer Interactions Help Explain the Connection between Stress and Cancer?

The NGF is released in humans following stress and social bonding [48]. The nerve–cancer interaction could be implicated in the theory that stress causes cancers, such as prostate cancer [49]. Hassan et al. [50] found that the epinephrine-activated b2-adrenergic receptor (ADRB2) increased the resistance of prostate cancer xenografts to cytotoxic therapies. Kim-Fuchs et al. [51] found that stress caused by confining mice to a confined space caused primary cancer growth and metastasis. Palm et al. described that norepinephrine, associated with stress, can induce prostate cancer metastases in vitro, while propranolol inhibited this effect [51].

Mindfulness-based cognitive therapy (MBCT) has therapeutic potential for the nerve–cancer relationship. This is cognitive therapy with meditative practices. In a randomized study of depressed patients in the arm treated with MBCT, both brain-derived neurotrophic factor (BDNF), a neurotrophin important for neuronal survival, growth, and maintenance in key brain circuits, and NGF were significantly decreased compared with the controls [52].

Another therapeutic potential is the use of beta-blockers. It has been shown that stress-related signaling through the adrenergic and glucocorticoid pathways can induce cell proliferation in human prostate cancer tissue [53]. In mice, stress has been shown to promote prostate carcinogenesis through adrenaline and the activation of the b2-adrenergic receptor (ADRB2), and behavioral stress can inhibit apoptosis [54]. Lu et al. [55] found that the activation of the adrenergic pathway was statistically significantly associated with lethal prostate cancer in the physicians’ health study and the health professionals’ follow-up study.

## 4. What Is Known about the Interaction between Nerves and Cancers in PCa and PDAC?

### 4.1. Crosstalk in Prostate Cancer

The prostate is richly innervated by both sympathetic and parasympathetic nerves, which help in both growth control and homeostasis [56]. Table 2 lists some clinical evidence and the effect of the interaction between cancer and nerves.

In prostate cancer, it has been shown that newly formed autonomic nerve fibers grow into cancer and thereby contribute to cancer initiation and progression through the activation of β-adrenergic and muscarinic cholinergic signaling [1].

It has been shown that veterans with spinal cord injury (SCI) had a much lower risk of PCa than age-matched controls [65].

There were fewer prostate cancers in the SCI group with complete motor injury. There was no correlation between the anatomical level of SCI and the prevalence of prostate cancer. This was a small study with only 350 veterans with SCI, with only seven men developing PCa; however, this was the only study in humans showing that denervation could have an influence on prostate cancer.

### 4.2. Denervation as a Therapeutic Approach

The therapeutic potential of denervation has been previously described. In a rat model of breast cancer, radical and persistent denervation resulted in cancer regression and long-term survival, whereas untreated rats died very quickly [34]. Magnon et al. [58] investigated the effects of sympathectomy on prostate cancer in a mouse model. Eleven weeks after the injection of prostate cancer cells into the mouse prostate, tumor-infiltrating sympathetic nerve fibers were found. Chemical sympathectomy prevents the development of prostate cancer.

### 4.3. Radiation Therapy as a Therapeutic Approach

Radiation therapy is an alternative to chemical and surgical nerve ablation. High radiation doses can have quasi-surgical effects on many tissues.

In a prospective single-arm multicenter phase two clinical trial, it was shown that patients treated with a single fraction of 25 Gy to the celiac plexus who had a better pain response also had longer survival. Such a high single dose can be considered as neuroablative [66].

### 4.4. Beta-Blockers as a Therapeutic Approach

There seems to be therapeutic potential for influencing beta-receptors. It has been shown that beta-adrenergic signaling can induce neuroendocrine differentiation in prostate cancer.

Prostate cancer is an interesting subject for studying crosstalk. It is not known whether this beneficial effect of local therapy is due to an effect on the nerves in the prostate. The above-mentioned positive results for surgical and neuroablation via radiation are in contrast to the clinical data for beta-blockers.

Beta-blockers have high therapeutic potential for cancer. Beta-adrenoceptors are the most frequent sympathetic receptors in human prostate tissue, with a dominance of the beta 2-subtype [67]. ADRB2 activation inhibits apoptosis and stimulates cell migration. The fact that nerves are implicated in prostate cancer has led several investigators to study whether alpha- or beta-blocker therapy has an influence on prostate cancer.

Alpha adrenoceptors mediate mainly smooth muscle contraction and vasoconstriction, while beta-receptors mediate, among other functions, vasodilation and smooth muscle relaxation [68]. However, the results have not been consistent. In a Finnish prostate cancer prevention trial, Murtola et al. [69] found that alpha-blockers did not reduce the overall prostate cancer risk; however, they found a decreased incidence of high-grade cancers (0.55; 95% CI 0.31–0.96). A meta-analysis conducted in 2015 [70] showed that beta-blockers are associated with reduced prostate cancer-specific mortality. Two meta-analyses found no association between beta-blocker use and overall survival or PCa mortality [71] or for any other antihypertensive drug [72]. These contradictory results could be due to the lack of specificity of the studied beta-blockers. A Norwegian study of cancer registry patients found that patients with non-selective beta-blockers that blocked both β1 and β2-adrenergic receptor (β2-AR) antagonists received less additional treatment after prostatectomy than patients treated with selective blockers that only blocked β1-AR [73]. Interestingly, β-receptors seem to be regulated by testosterone, at least in the rat prostate, with castration causing a decrease in β2-AR [67]. Despite the inconclusive data mentioned above, it seems possible that with a better understanding of the beta-receptors, one could influence the transformation to neuroendocrine cancers [53].

Another class of medications that could influence the nerve–cancer crosstalk is lidocaine, a local anesthetic that alters nerve signal conduction. In breast cancer surgery, the peritumoral injection of 0.5% lidocaine 7–10 min before surgery resulted in a small but significantly increased DFS and OS [74].

### 4.5. Perineural Invasion (PNI) in Prostate Cancer

The importance of PNI in prostate cancer has been extensively discussed. Niu et al. [75], in their recent 2022 review on the current knowledge of the prognostic significance of PNI in prostate cancer, concluded that PNI is commonly associated with adverse clinicopathological parameters and poor outcomes. However, the question remains as to whether PNI is an independent prognostic predictor. In an analysis of 696 men who underwent radical prostatectomy, Quinn et al. [76] found that PNI was present in 10.4% of prostate biopsies and 58.3% of RP specimens. PNI was an independent predictive factor for outcomes in patients with a PSA level >10 ng/mL. However, the presence of PNI in the specimen was not an independent predictor of extracapsular extension, seminal vesicle invasion, or metastasis.

Ayala et al. found an outgrowth of neurites into DU-145 cell colonies when the dorsal root ganglia (DRG) of the mouse, human prostate cancer cells (Du-145, LNCaP, and PC3) and stromal cells (HTS-40F) were co-cultured. DU-145 cells migrated along neurites into the nerve/ganglion [11].

### 4.6. The NGF in Prostate Cancer

Interestingly, for prostate cancer, the p75 NTR receptor is a low-affinity receptor to which all neurotrophins bind. This receptor promotes NF-κB, which is important for neuronal survival and induces metastasis, especially to the bone in prostate cancer, and is implicated in resistance to chemotherapy and radiotherapy [77]. The role of the NGF in prostate cancer has not been extensively studied. NGF receptors have been detected not only in central nervous system cancers but also in breast, lung, pancreas, and prostate cancers. In a recently published study analyzing the urinary NGF in patients with prostate cancer, the NGF was found to be predictive of the Gleason score, but the NGF was not correlated with PNI [78].

### 4.7. Crosstalk in Pancreatic Cancer

The neural network of the pancreas is complex and includes sympathetic, parasympathetic, and enteropancreatic fibers [28]. In PDAC, Schwann cells are activated by cancer [79]. Schwann cells then build tracks that enable cancer invasion. A clinical sign of this crosstalk was shown in a study in humans, showing that pancreatic sympathetic innervation was significantly reduced in patients with both chronic pancreatitis (CP) and pancreatic cancer (PCa), pointing to a “neural remodeling” in these diseases [80].

PNI is very common in PDAC and is an important prognostic factor [81]. The interdependence between the neural system and cancer cells in the pancreatic TME is a subject of research [2].

In patients with PDAC, antihypertensive drugs, including beta-blockers, appear to lack a clear antitumor effect [82], although there are few clinical and laboratory studies on beta-blockers [83].

Neuroablation has also been performed in patients with pancreatic cancer. In an animal model, the ablation of the sensory neurons that innervated the pancreas prevented neurogenic inflammation and delayed tumor formation in the pancreas [61].

### 4.8. The NGF in Pancreatic Cancer

It has been shown that pancreatic cancer cells with little serine can selectively increase the translation of the nerve growth factor (NGF), which, in turn, recruits axons as an exogenous source of serine. Preclinical research has shown that there is axonal–cancer metabolic crosstalk that is important for ductal pancreatic carcinoma growth [6,84].

Chronic stress can induce norepinephrine in PDAC through sympathetic innervation [85]. This upregulates the expression and secretion of the NGF and the brain-derived neurotrophic factor (BDNF). This, in turn, accelerates tumorigenesis and leads to reduced survival in mice.

## 5. Conclusions

Bidirectional crosstalk between the nervous system and cancer cells has emerged as a crucial regulator of cancer and its microenvironment. The neural axis influences cancer progression via the autonomous nervous system. Although medical therapy, such as beta-blockers, does not seem to have an influence on neurotropic cancers such as prostate cancer, surgical denervation has been shown to influence mammary cell lines in rats. A recent clinical study of neuroablative doses to the celiac plexus reported a better pain response and longer survival. At the same time, it has been recognized that there is a potential relationship between cancer and psychosocial biology via neurotransmitters and neurotrophins; however, the biological pathways are largely unknown.

## Figures and Tables

**Table 1:** General evidence of an interaction between cancer and nerves.

Author	Finding	Reference
Hanahan	Innervation of tissue stem cell niches	[27]
Hanahan	Innervation causes resistance against cell death	[27]
Mauffrey	Axonal outgrowth for interaction with the TME with the regulation of tumorigenesis and metastasis	[1]
Monje	Direct and indirect nerve–cancer crosstalk interactions with immune and endothelial cells	[8]
Tan	Cancer-associated fibroblasts produce matrix metalloproteases that regulate neural development	[28]
Tan	Production of stellate cells favors nerve outgrowth during tumor development in the tumor microenvironment (TME)	[28]

**Table 2:** Clinical evidence and effect of the interaction between cancer and nerves.

Author	Finding	Reference
Hanahan	Glutamatergic neuronal activity can drive proliferative signaling in certain central nervous cancers	[57]
Magnon	Adrenergic signaling from sympathetic nerves in the TME promotes tumorigenesis	[58]
Magnon	Parasympathetic muscarinic receptors regulate tumor invasion and metastasis	[58]
Mitsou	Surgical denervation led to long-term survival in a rat model	[34]
Coarfa	Chemical denervation with Botox caused apoptosis in a mouse model	[59]
Guo	Perineural invasion is present in many different cancers and associated with cancer invasion, recurrence, and metastasis	[60]
Saloman	The capsaicin-induced ablation of the innervation of the neonatal pancreas causes a delay in tumor formation	[61]
Magnon	Axonogenesis in prostate cancer can contribute to cancer growth and dissemination in a mouse model	[58]
Campell	Adrenergic signaling activates osteoblasts that favor bone metastasis	[62]
Griffin	Cancer cells can release the NGF, which influences tumor neurogenesis. Nerves infiltrate the TME and secrete neurotransmitters, which stimulate tumor cell growth and angiogenesis	[63]
Blondy	Neurotrophins are key messengers in the crosstalk between cancer cells and peripheral nerve fibers	[64]
